# Electric Field Comparison between Microelectrode Recording and Deep Brain Stimulation Systems—A Simulation Study

**DOI:** 10.3390/brainsci8020028

**Published:** 2018-02-06

**Authors:** Fabiola Alonso, Dorian Vogel, Johannes Johansson, Karin Wårdell, Simone Hemm

**Affiliations:** 1 Department of Biomedical Engineering, Linköping University, 58185 Linköping, Sweden; dorian.vogel@fhnw.ch (D.V.); johannes.johansson@liu.se (J.J.); karin.wardell@liu.se (K.W.); simone.hemm@fhnw.ch (S.H.); 2 Institute for Medical and Analytical Technologies, School of Life Sciences, University of Applied Sciences and Arts Northwestern Switzerland FHNW, 4132 Muttenz, Switzerland

**Keywords:** microelectrode recording (MER), finite element method (FEM), deep brain stimulation (DBS), brain model, Dice coefficient, patient-specific

## Abstract

The success of deep brain stimulation (DBS) relies primarily on the localization of the implanted electrode. Its final position can be chosen based on the results of intraoperative microelectrode recording (MER) and stimulation tests. The optimal position often differs from the final one selected for chronic stimulation with the DBS electrode. The aim of the study was to investigate, using finite element method (FEM) modeling and simulations, whether lead design, electrical setup, and operating modes induce differences in electric field (EF) distribution and in consequence, the clinical outcome. Finite element models of a MER system and a chronic DBS lead were developed. Simulations of the EF were performed for homogenous and patient-specific brain models to evaluate the influence of grounding (guide tube vs. stimulator case), parallel MER leads, and non-active DBS contacts. Results showed that the EF is deformed depending on the distance between the guide tube and stimulating contact. Several parallel MER leads and the presence of the non-active DBS contacts influence the EF distribution. The DBS EF volume can cover the intraoperatively produced EF, but can also extend to other anatomical areas. In conclusion, EF deformations between stimulation tests and DBS should be taken into consideration as they can alter the clinical outcome.

## 1. Introduction

Deep brain stimulation (DBS) is an established surgical therapy to treat the symptoms from movement disorders such as Parkinson’s disease, essential tremor, and dystonia [1,2,3]. The electrical stimulation affects a population of neuron axons in the vicinity of the active electrode. The extension in space of the stimulation depends on several factors, including the surrounding tissue properties, the electrode design, and the stimulation parameters [4].

The success of the surgery is highly dependent on the correct electrode placement, which requires the utmost accuracy in the targeting stage. Ideally placed electrodes results in symptom removal at low stimulation amplitudes and higher thresholds for stimulation-induced side effects.

Due to difficulties in the visualization of some structures with conventional magnetic resonance imaging (MRI), a common procedure to confirm or refine the localization of the target before the insertion of the permanent DBS lead, is to monitor the deep brain structures along the pre-planned trajectory through a microelectrode recording (MER) system. The MER lead consists of a microelectrode at the tip used to record the spontaneous electrical activity along a pre-planned trajectory down to the target, and a macroelectrode to perform functional stimulation tests for clinical assessment of the patient’s symptoms.

Clinical experience [5,6,7] shows that the position of the DBS contact finally activated for chronic stimulation to induce the best clinical outcome often differs from the intraoperatively chosen position. Possible reasons may include brain shift or the subjective interpretation of stimulation test results.

Computer models using FEM [8,9] have been widely used to investigate the influence of the stimulation parameters and surrounding tissue properties in DBS. The electric field (EF), i.e., the potential’s first derivative, can be used to approximate the volume of tissue activated (VTA) for specific stimulation parameters and axon diameters, and thus the volume within a fixed EF isolevel [10]. The EF isolevel approach has been used by numerous studies [11,12,13,14,15,16,17,18] and allows for presentation of the results in the millimeter scale and thus for relative comparisons between simulations. To our knowledge, a comparison between intraoperative stimulation using the MER macroelectrode and chronic stimulation using a standard DBS lead has not yet been addressed through computer models.

The aim of this study was to compare and evaluate the electric field (EF) distributions of the MER macroelectrode and DBS lead assuming that they are positioned along the same trajectory. Differences in lead design, operating modes, and electrical setup were investigated by means of FEM models and simulations.

## 2. Materials and Methods

FEM simulations performed in this investigation are based on the surgical protocol from the Department of Neurosurgery at Clermont-Ferrand University Hospital, where intraoperative MER and stimulation tests are performed prior to DBS lead implantation [16].

### 2.1. Intraoperative Stimulation and DBS Implantation

**Figure 1:**
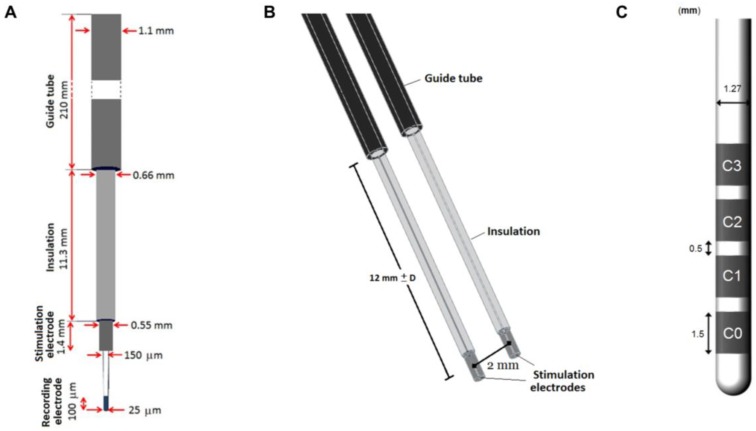
Schematic representation of the microelectrode recording (MER) system and DBS. (**A**) The intraoperative MER lead, including the guide tube and recording electrode. (**B**) 3D model including a parallel lead in the posterior trajectory along the preoperative planned trajectory and (**C**) DBS lead 3389 model.

During surgery, one or up to five MER leads are inserted along trajectories according to the preoperative MRI-based planning. Neural activity of the region of interest is recorded with the MER tip followed by stimulation tests using the MER stimulation contact (Figure 1A). The MER lead (Neuroprobe 366-000024, Alpha Omega Engineering, Nazareth, Israel) is steered to the target region through a rigid guide tube (ACS-7905/200-5, DIXI Microtechniques, Besançon, France). The distal end of the guide tube is inserted as deep as 12 mm before the planned target point, and fixed at that position (Figure 1). When more MER leads are used, guide tubes are placed in parallel, 2 mm from each other (Figure 1B). Before the intraoperative stimulation tests are performed, a first path is made to record the activity with the MER tip along the pre-planned trajectory; afterwards, the MER tip is retracted some millimeters inside the stimulation macroelectrode. The stimulation tests are performed in the range between 10 mm before the planned target and 4 mm beyond it, in 1 mm steps. A cathodic monopolar configuration is used to apply constant current pulses through the MER stimulation contact of one of the inserted leads while the parallel one remains off. The anode or reference electrode is set to the guide tubes. A detailed description of the procedure can be found in [16].

The DBS lead (3389, Medtronic Inc., Minneapolis, MN, USA) is subsequently implanted at the location where the highest therapeutic effect is achieved with minimal stimulation amplitudes and side effects with the MER stimulation tests.

### 2.2. Finite Element Method Models

The FEM models were designed in accordance with the clinical configuration for the intraoperative and chronic stimulation. Homogeneous 2D axisymmetric and patient-specific 3D models were developed with COMSOL Multiphysics (Ver. 5.2, COMSOL AB, Stockholm, Sweden) to investigate the differences between the intraoperative and DBS electrodes in terms of the EF distribution.

The electric field magnitude, EF = |−∇V|, around the electrode was calculated by the equation for steady currents,

(1) $$\nabla \cdot J=-\nabla \cdot \left(\sigma \nabla V\right)=0\;A/m^{3}$$

where ∇ is the divergence, J (A/m^2^) is the current density, σ (S/m) is the electrical conductivity, and V (V) is the electric potential.

#### 2.2.1. MER Lead Model

The geometry of the intraoperative lead model corresponds to the Neuroprobe. The recording tip was not considered in the model as it is retracted during stimulation. The guide tube was placed 12 mm apart from the middle point of the stimulation contact and fixed at that position (Figure 1B).

The MER stimulation contact was set to a constant current. The non-active parallel contacts were set to floating potential (∫−n·σ∇VdS=0 A; n×(−∇V)=0 V/m), while the non-conductive shaft was set to insulation (n·∇V=0 V/m), where **n** is the surface normal vector. The guide tubes were considered as the anode and were set to ground (V = 0 V).

#### 2.2.2. DBS Lead Model

The geometry of the DBS lead corresponds to the model 3389, surrounded by a 250 μm thick peri-electrode space (PES) in order to mimic the fibrous tissue developed over time after the lead is implanted. The PES was assumed to have the same electrical conductivity as white matter, σ = 0.075 S/m [19,20]. Monopolar configuration was simulated by setting the active contact to a constant voltage and the outer boundaries of the surrounding medium to ground. The non-active contacts were set to floating potential.

#### 2.2.3. Brain Models

Three brain models were created: a 2D axisymmetric homogenous, 3D homogenous, and 3D heterogeneous (patient-specific) model. The surrounding medium of the contact for the 2D axisymmetric (54 × 100 mm^2^) and 3D models (100 × 154 × 90 mm^3^) was homogeneous and isotropic, with an electrical conductivity of 0.123 S/m corresponding to grey matter.

The 3D heterogeneous model in turn considered a medium corresponding to patient-specific data, obtained from a preoperative stereotactic T1-weighted MRI (1.5 Tesla, Sonata, Siemens GmbH, Munich, Germany) performed with a Leksell^®^ G frame (Elekta Instrument AB, Stockholm, Sweden) mounted to the patient’s head [9,14].

An MRI (0.63 × 0.63 × 1.30 mm^3^) batch was used from a patient suffering from essential tremor who had been implanted in the ventral intermediate nucleus (Vim) of the thalamus (written consent has been obtained from the patient; ethic approval ref.: 2011-A00774-37/AU905, Comité de Protection des Personnes Sud-Est 6, Clermont-Ferrand, France; approval date: 21st of July 2011) to obtain the brain model, suitable for FEM simulations. The brain model is an interpolation matrix of the corresponding electrical conductivity of the relevant tissue types, i.e., gray matter (0.123 S/m), white matter (0.075 S/m), cerebrospinal fluid (2.0 S/m), and blood (0.7 S/m) [21,22]. The conductivity values considered a pulse width of 60 µs and a pulse frequency of 130 Hz.

The mesh was physics-controlled with a denser distribution around the leads. For the 2D model, the mesh consisted of approximately 17,000 triangular elements. The minimum element size was 0.002 mm with an average element quality of 0.9. For the 3D models, the mesh consisted of approximately 3,000,000 tetrahedral elements with a minimum element size of 0.03 mm and an average element quality of 0.7.

The leads for the 3D models were positioned based on the patient-specific clinical data, i.e., planned trajectory and target coordinates (Figure 1B).

### 2.3. Simulations

The boundary conditions in the FEM models, stimulation settings, and relative position between the leads were modified in order to investigate the influence of the electrode design, operating mode, and electrical setup. A clinical case was explored to observe differences on the EF obtained between the patient-specific intraoperative vs. postoperative stimulation settings. Simulations were run for clinically relevant stimulation amplitudes (1, 2, 3, or 4 V) for the DBS and 1, 2, 3, or 4 mA for the MER stimulation contact, which achieves the same EF extension using the described 2D homogeneous model.

#### 2.3.1. Grounding the Guide Tube

During the intraoperative stimulation tests, the guide tube is used as the reference electrode (anode) and is set to ground, in contrast to the chronic DBS stimulation, where the ground is set to the neurostimulator case. The 2D axisymmetric model was used to explore how the EF is influenced by the grounded guide tube. Simulations were performed once with the guide tube grounded and once with modified boundary conditions for the intraoperative lead by setting the guide tube to floating potential while the ground was set to the outer boundaries of the medium. The MER stimulation contact was placed at the target (12 mm from the guide tube), and set from 1 to 4 mA in 1 mA steps. Simulations were also performed displacing the MER stimulation contact up to 10 mm above and 4 mm below the target, i.e., 2 and 16 mm from the guide tube, respectively, in 1 mm steps.

#### 2.3.2. Parallel MER Leads

When more than one lead is inserted during the stimulation tests, the metallic contacts of the parallel leads may affect the EF distribution. The homogenous 3D model was used to compare and evaluate the EF for different scenarios: (a) one single lead (central trajectory); (b) two parallel leads (central and posterior trajectories); and (c) three parallel leads (central, posterior, and medial trajectories). The parallel leads, with identical geometry and material properties, were placed 2 mm posterior, and medially to the central lead. The non-active MER stimulation contacts were set to floating potential and simulations were run setting the active contact to 1 and 3 mA.

#### 2.3.3. Non-Active Contacts for DBS

While the MER lead consists of only one stimulation contact, the presence of non-active contacts on the DBS lead becomes important to assess since they may affect the EF distribution around the lead. The homogeneous 2D model was used to evaluate the EF by activating each of the contacts (C0, C1, C2, C3) (Figure 1C) while setting the non-active ones to floating potential. The DBS lead was displaced along its axis in order to align the active contact to the MER contact, using the middle point of each contact as a reference. The EF was also compared for a fixed position of the DBS lead aligning the first contact, C0, to the target position and activating each of the DBS contacts (C0, C1, C2, C3) separately.

#### 2.3.4. Position of the DBS Active Contact

Simulations were also performed with the patient-specific 3D model in order to consider the heterogeneity of the surrounding medium.

In order to investigate to which extent the EF induced by the MER stimulation can be reproduced by the DBS contacts in different positions, the DBS lead was displaced along the same trajectory as the MER lead so that the middle point and the lower and upper edges of the active DBS contacts were placed consecutively at the target position.

#### 2.3.5. Clinical Case

The patient-specific 3D brain model and the clinical settings were used to compare the EF generated by each contact. The position of the MER lead was based on the preoperative MRI planned trajectory to target the Vim. The stimulation contact of the MER central lead was positioned 2 mm beyond the target and was set to 0.4 mA, corresponding to the stimulation position and amplitude where the best symptom improvement (94%) was achieved [23]. The posterior trajectory contact was set to floating potential. The DBS lead, in turn, was positioned according to the coordinates taken from the postoperative CT scan after image co-registration with preoperative stereotactic MRI. The third contact (C2), set to 2 V, was used for chronic stimulation (80% improvement) programmed six months after surgery. Simulations were also performed for the first (C0) and second (C1) contact at the same stimulation amplitude and 3 V.

### 2.4. Data Analysis

The EF was visualized and measured using a 0.2 V/mm isolevel for all the cases investigated [10,16]. For the 2D models, the maximum radial extension of the EF isocontour was measured from the lead axis. Differences between the investigated scenarios were visually assessed by superimposing the corresponding EF distribution for each situation. For the 3D patient-specific simulations, the volume within the 0.2 V/mm isosurface was calculated, i.e., the VTA. The volumes for the DBS and the MER lead were compared by calculating the Sørensen-Dice coefficient (DC) [24]. The volume overlap between two structures was rated according to Equation (2),

(2) $$\mathrm{DC}=2\frac{\left|V_{\mathrm{MER}}\cap V_{\mathrm{DBS}}\right|}{\left|V_{\mathrm{MER}}\right|+\left|V_{\mathrm{DBS}}\right|}$$

where V_MER_ corresponds to the volume obtained for the MER system stimulation electrode and V_DBS_ for the implanted DBS lead (vertical bars indicating a summation of the included voxels).

A perfect overlap between two volumes results in a value of 1 for the coefficient, decreasing to DC = 0 when no common volume between the two exists.

V_MER_ and V_DBS_ were calculated by exporting the results of FEM simulations as polygonal meshes and were processed using filters from the VTK toolkit (vtk.org, ver. 7.1, Kitware Inc., Clifton Park, NY, USA), assembled in a Paraview pipeline (paraview.org, ver. 5.4, Kitware Inc., USA). Polygonal (closed) surfaces of electric fields and their respective electrode volume were sampled to binary mask images using a 0.1 × 0.1 × 0.1 mm^3^ voxel size. Electrode volume was removed from each corresponding EF using boolean image operations, and both the intersection and union were calculated for each EF permutation. The volume of each resulting mesh volume was then extracted and used to calculate the DC. Together with the DC, the MER EF Coverage Coefficient (CC) was used, expressed as the volume of the intersection of the two volumes normalized to the volume of the MER electric field, according to Equation (3)

(3) $$\mathrm{CC}=\frac{\left|V_{\mathrm{MER}}\cap V_{\mathrm{DBS}}\right|}{\left|V_{\mathrm{MER}}\right|}$$

A summary of all simulations is presented in Table 1.

## 3. Results

### 3.1. Grounding the Guide Tube

**Figure 2:**
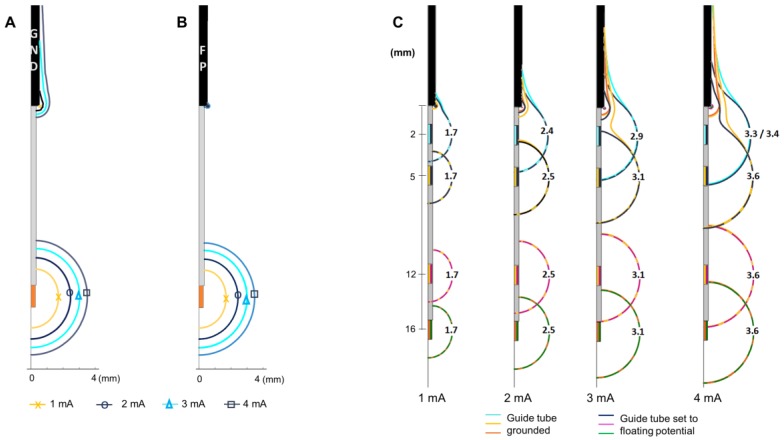
Influence of grounding the guide tube. Electric field (EF) isocontours (0.2 V/mm) simulated in homogeneous grey matter setting the MER stimulation contact to 1 to 4 mA in 1 mA steps. (**A**) Guide tube at target position set to ground (GND), (**B**) guide tube set to floating potential (FP), outer boundaries of the model set to ground. (**C**) EF isocontours overlapped to compare the EF when the guide tube is grounded and set to floating potential. EF simulated placing the MER electrode at different distances from the guide tube (distance to target: +4 mm, 0 mm, −7 mm, −10 mm) exemplified for amplitudes between 1 to 4 mA. Numbers in front of the active contact indicate the maximum distance of the isocontour to the lead axis.

The main influence of grounding the guide tube is the presence of an EF around it. The EF around the guide tube increases with the stimulation amplitude when the guide tube is grounded (Figure 2A) in contrast to the negligible EF when the guide tube is set to floating potential (Figure 2B). The EF around the active contact is not visibly modified at any stimulation amplitude. The maximal extension of the 0.2 V/mm isocontour is the same when the guide tube is grounded or in floating potential.

The EF distribution around the active contact is affected at shorter distances to the guide tube (stimulation contact 10 mm above the target) for both cases; grounded and floating potential. The EF loses its spherical shape and is deformed towards the guide tube for all the stimulation amplitudes investigated (Figure 2C). The EF around the active contact does not present any change for distances longer than 6 mm between the stimulation contact and the guide tube (Figure 2C) for the selected amplitudes.

### 3.2. Parallel MER Leads

**Figure 3:**
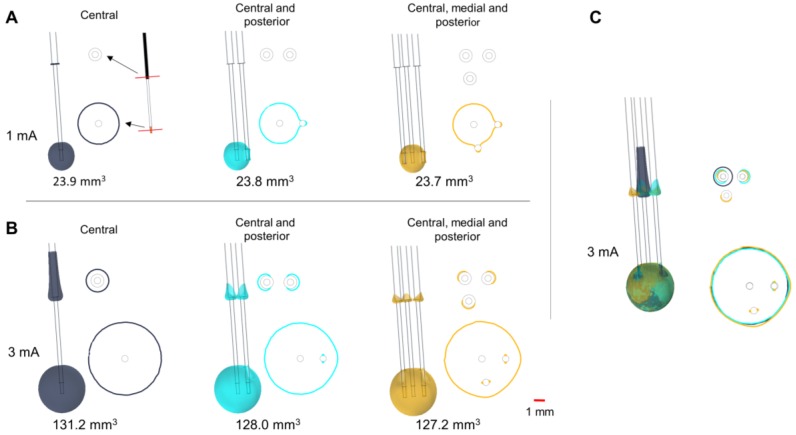
Influence of parallel MER trajectories inserted intraoperatively. EF distribution simulated in a homogeneous medium depicted with isosurfaces and isocontours of 0.2 V/mm. Isocontours obtained at perpendicular planes placed at the target and 12.5 mm above it (red lines shown in A) for stimulation using the central lead at (**A**) 1 mA and (**B**) 3 mA. (**C**) Superposition of the isosurfaces and the isocontours for the three cases at 3 mA. Single lead (dark blue), two leads (cyan), and three leads (yellow). Non active contacts of the posterior and medial leads were set to floating potential. Numbers below the leads indicate the EF volume covered by the 0.2 V/mm isosurface.

The insertion of parallel leads changes the EF around the guide tubes (Figure 3), which is especially visible at high amplitudes (Figure 3B). The EF extension along the central guide tube decreases when adding parallel leads, while the horizontal extension between the different guide tubes increases. Isocontours obtained at the level of the active contact show the presence of an EF around the parallel non-active contacts, which slightly changes the EF extension and shape (Figure 3A–C).

### 3.3. Non-Active Contacts for DBS

**Figure 4:**
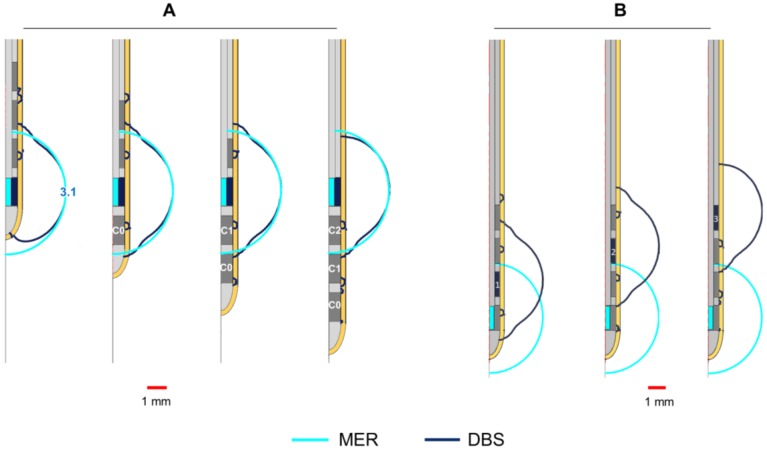
Influence of non-active contacts for DBS. EF simulated in homogeneous grey matter (0.123 S/m) depicted with 0.2 V/mm isocontours. (**A**) Activation of each DBS contact displacing the DBS lead to align the middle point of the active contact (dark blue) to that of the MER stimulation contact (cyan). Numbers in front of the active contact of the first lead indicate the maximal EF extension measured from the lead’s axis. (**B**) Activation of each DBS contact while the first contact (C0) is aligned to the middle point of the MER stimulation contact. The DBS contacts were set to 3 V one at the time and the MER stimulation contact was set to 3 mA. Non-active contacts (in dark grey) were set to floating potential. Maximal extension obtained for the MER and DBS contacts: 3.1 mm.

The shape of the EF around the DBS lead is modified at all stimulation amplitudes compared to the spherical distribution around the MER contact, due to the adjacent non-active contacts. Despite the deformation, the maximal extension of the EF isocontours (Figure 4A,B) is nearly the same (±0.03 mm) for each contact at any amplitude: 1.7, 2.5, 3.1, and 3.6 mm for 1, 2, 3, and 4 mA or V, respectively.

### 3.4. Position of the DBS Active Contact

**Figure 5:**
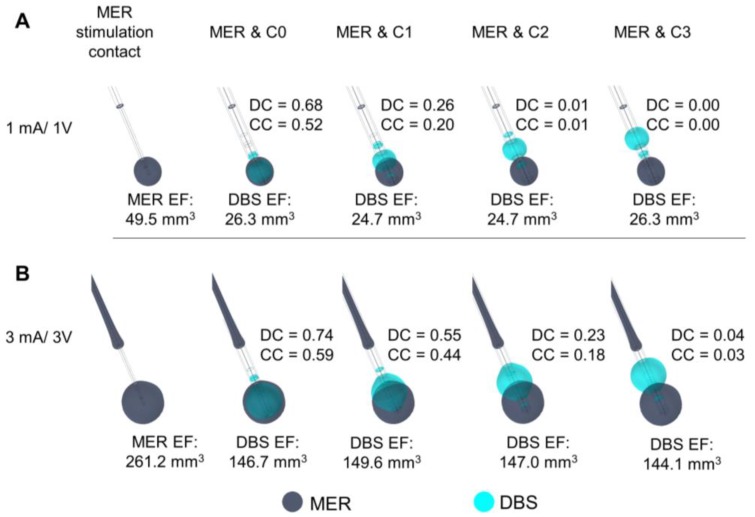
Comparison of the EF between the MER stimulation contact and each DBS contact simulated in a heterogeneous medium (σ ≈ 0.079 S/m). DBS first contact (C0) (cyan) aligned to match the middle point of the MER stimulation contact (dark blue). EF isosurfaces (0.2 V/mm) superimposed for (**A**) MER stimulation contact set to 1 mA and the active DBS contact set to 1 V, and (**B**) for 3 mA and 3 V. EF volume within the selected isosurface shown below the lead and Dice coefficient (DC) and coverage coefficient (CC) shown to the right of each lead.

**Figure 6:**
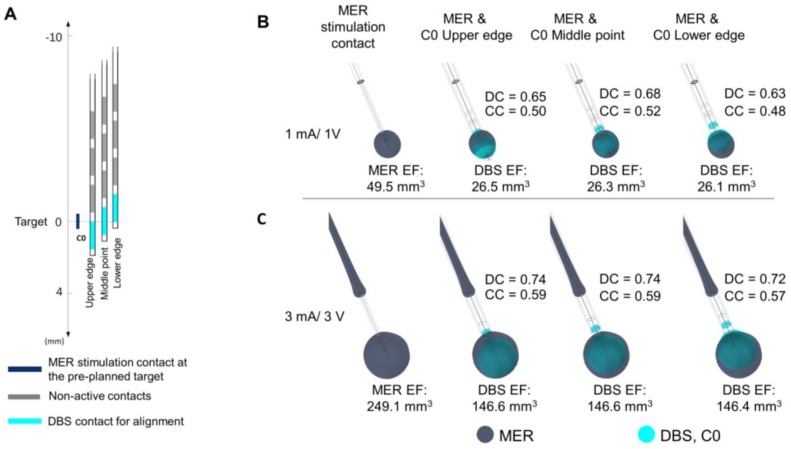
Evaluation of the position of the DBS contact in heterogeneous tissue (σ ≈ 0.079 S/m). (**A**) Displacement of the DBS lead to match the lower edge, the middle point, and the upper edge to the middle point of the MER stimulation contact localized at the target. Overlay of the EF isosurfaces (0.2 V/mm) simulated for the MER stimulation contact (dark blue) and the DBS first contact C0 (cyan) set to (**B**) 1 mA and 1 V and (**C**) 3 mA and 3 V, respectively. EF volume within the selected isosurface shown below the lead. DC and CC shown to the right of each lead.

The comparison between EF for the MER stimulation and each DBS contact is presented in Figure 5. The highest DC and CC are set to 3 mA/3 V for C0. An EF overlap between the MER stimulation contact and DBS is possible for amplitudes higher than 3 V, even when the position of the contacts differs by 4 or 6 mm (Figure 5B). The highest DC reflects the best match in the case where the DBS contact is aligned to the MER stimulation contact. When comparing differences in EF for alignments of the upper and lower edge and the center of contact C0 in relation to the center of the MER contact, the best match of the EF volumes is also obtained for an alignment of the two middle points at any stimulation amplitude (Figure 6). The DC reveals, however, that it is possible to have a high index of coincidence at high amplitudes when the two contacts are not aligned, as shown for 3 mA/3V in Figure 6C. A higher coverage coefficient is consistently obtained for higher amplitudes (3 mA) in comparison to lower amplitudes (1 mA).

A larger EF volume was obtained for the MER stimulation contact in comparison to the homogeneous case (Figure 3, one lead). The electrical conductivity for the patient- specific model was averaged within a cubic region (10 mm^3^) centered at the target resulting in 0.079 S/m, which is a low value compared to the homogeneous model (0.123 S/m). In order to deliver a constant current of 3 mA through the MER stimulation contact, the lower conductivity of this patient-specific model required a voltage of 9.8 V in comparison to the homogeneous grey matter where 6.0 V was applied to obtain the same current.

### 3.5. Clinical Case

**Figure 7:**
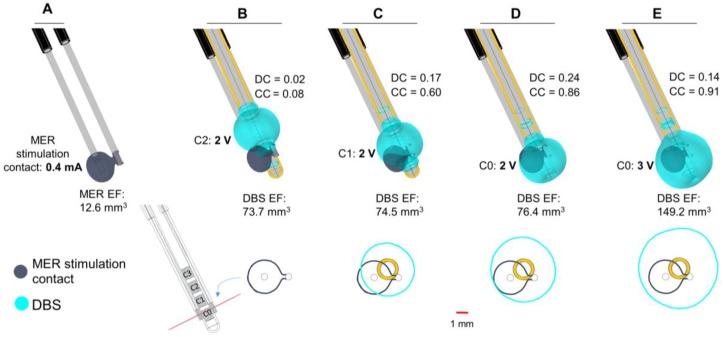
Clinical case. Electric field distribution depicted with 0.2 V/mm isosurfaces (upper panel) and isocontours (lower panel) obtained at the perpendicular plane placed at the middle point of the MER stimulation contact (red line). MER and DBS lead trajectories obtained from the preoperative plan and the postoperative CT scan, respectively. (**A**) Stimulation tests performed with the MER stimulation electrode placed at the optimal position determined intraoperatively: 2 mm beyond the pre-planned target on the central trajectory with a stimulation amplitude of 0.4 mA. (**B**) Overlap of the EF obtained with the MER stimulation electrode (dark blue) and the DBS contact C2 (cyan) set to 2 V, programmed six months after surgery. DBS lead placed 3 mm beyond the target (lower edge of the first contact, C0); (**C**,**D**) Additional comparisons using DBS contacts C1 and C0 set to 2 V and (**E**) C0 set to 3 V. EF volume within the selected isosurface shown below each lead. DC and CC shown to the right of each case.

In the presented case, the trajectory and the final position for the implanted DBS lead deviated from the intraoperative MER lead. The EF isosurface using the MER stimulation electrode is smaller compared to the EF obtained setting the DBS electrode to the stimulation amplitude programmed six months after surgery (Figure 7B). The EF isocontours obtained at the perpendicular plane placed at the midpoint of the MER stimulation contact show no overlap at the optimal spot (Figure 7B, lower panel) and a very low DC value. With the same amplitude of 2 V, the activation of DBS contacts closer to the MER stimulation contact increases the coincidence of the EF, and thus the DC (Figure 7C,D). A complete coverage of the intraoperative EF is achieved with a higher amplitude (3 V) using contact C0, the closest DBS contact to the MER (Figure 7E), consequently the large value of the coverage coefficient. The DC, however, is lower than for the other cases investigated.

## 4. Discussion

The main objective of the present study was to investigate, by means of FEM models and simulations, differences in the EF distribution between MER stimulation and DBS which could explain variations in clinical outcome. The study was focused on the evaluation and visualization of the EF since it is the electrical entity directly affected by changes in the lead design, operating mode, and electrical setup. Other studies have addressed the influence of the electrode geometry, impedance, and other aspects using evaluation parameters such as the current density or the VTA [25,26,27]. Those studies, however, examine a single DBS lead or electrode under voltage controlled stimulation. The present investigation, in contrast, compares two electrodes with differences in their operating mode, setup, and dimensions. For this comparison, the specific value of 0.2 V/mm isolevel has been used. The selection of the 0.2 V/mm is based on the results from previous studies [10,14,28] which have investigated the stimulation field combining FEM and neuron models. The 0.2 V/mm isolevel is the required EF magnitude to stimulate neurons of around 3–4 µm with a pulse width between 60 to 90 µs. Moreover, this isolevel has been used in other FEM-simulation studies [13,18] and represents a useful value for comparative purposes.

### 4.1. Electrical Setup

The results regarding the influence of the grounded guide tube have shown that the 0.2 V/mm isosurface is present not only around the stimulating contact, but also around the guide tube. This raises the question of whether neuronal activation is possible in those regions, which are not intended to be stimulated. Further studies are necessary to investigate the occurrence of this effect.

Furthermore, the proximity to the grounded guide tube has been shown to influence the form of the EF around the stimulating contact when approaching the guide tube. The importance of this deformation increases with the increase of the stimulation amplitude. Depending on the anatomical position, such deformations might be responsible for some side effects. Even if the optimal stimulation area where the DBS lead is finally implanted is in general somewhere around the chosen anatomical target, i.e., more than 5 mm away from the guide tube, the medical staff should be aware of those differences and consider further retracting the guide tube in those regions.

The presence of the parallel leads during MER also affects the EF distribution. A deformation of the EF in the lateral direction at the level of the stimulating contact and especially of the guide tube exists compared to the use of only one trajectory. Nevertheless, a neuronal activation at the level of the guide tube might still be possible.

### 4.2. Non-Active Contacts for DBS

The investigation of the influence of the non-activated contacts of the DBS lead, placed in the same position as the MER contact, shows that the EF extension obtained for the MER stimulation contact is quite reproducible with the DBS contact. The highest but still slight difference could be seen for contact C0 with a lower extension down the trajectory. For clinical practice, this means that to limit the influence of this effect and to guarantee the best reproducible form, another contact other than C0 could be put at the position with the best clinical result obtained during intraoperative stimulation.

### 4.3. Position of the DBS Active Contact

The present study also demonstrated that it is possible to have an overlap of EF between the MER and the DBS, even when the active contacts are not in the same location (Figure 4, Figure 5 and Figure 6). Nevertheless, to reproduce the same EF extension as with the MER electrode, the center of a DBS contact should be optimally placed at this position as the different DC and CC have shown. At other positions, it is also possible to partially or completely cover the volume generated by the MER lead, but the consequence might be a non-necessary extension in other directions as seen by lower DCs, which could induce side effects and which result in a higher energy consumption.

### 4.4. Clinical Case

The clinical application shows an example of a patient where the final stimulation position with the DBS lead does not correspond to the intraoperatively identified position, which produced the best clinical effect (Figure 7). The simulations, performed by placing the DBS lead at the coordinates identified on the postoperative CT images, showed no overlap of the EF at the level of the optimal position and a very low Dice coefficient. The DBS contact selected and programmed six months after surgery, however, achieved a satisfactory improvement in the relief of the patient symptoms. Plausible reasons for differences in the MER and DBS trajectories may include imaging uncertainties or brain shift. The hypothetical cases presented in Figure 7D,E suggest a better selection of chronic stimulation according to the EF distribution and a higher coverage coefficient. The application of the presented approach to numerous patients and the correlation with the anatomical positions would be very interesting in terms of further analyzing differences of intraoperative and chronic stimulation.

In order to base the simulations on realistic examples, the chosen electrode types and parameters originate from the clinical DBS protocol at the Department of Neurosurgery, Clermont-Ferrand University Hospital [16]. One has to be aware that there may be differences in protocols depending on the chosen MER system and DBS implantation procedure at the individual clinical center. Some clinics abandon MER and others use the chronic DBS lead for intraoperative tests in a bipolar or monopolar configuration. The monopolar test stimulation uses the addition of a contact, either as a guiding cannula or a scalp needle, to close the current loop substituting the neurostimulator as it is not implanted at this point. The FEM simulations performed in this study do not consider all possible situations, but give an idea of potential influencing factors that the medical staff should keep in mind. As presented in Figure 7, the DBS lead will most likely not be able to exactly reproduce the EF generated by the MER setup. By presenting this general method for modeling and simulation, additional studies for similar clinical protocols and MER-systems can be studied in the future.

### 4.5. Methodology

The size of the MER and DBS contacts varies, especially in the lateral direction (0.55 mm vs. 1.27 mm). For the 3D models and the DC calculation, the contact volumes have been considered as described in the method section. Nevertheless, the results of the 2D simulations (Figure 4) have to be interpreted with care as the extension has been determined starting from the center line of the electrodes and not from the surface. As the lateral dimension of the MER electrodes is smaller than the one of the DBS lead, the volume of tissue included for the same extension will be higher for MER stimulation than for DBS.

The DC quantifies the similarity between two volumes; however, the ultimate goal for the DBS electrode implantation is to reproduce the improvement observed during MER exploration. To this extent, CC gives a representation of up to how much DBS stimulation can potentially reproduce the effect of the MER stimulation. However, tissue stimulated during DBS, which was not included in the MER stimulation, can be the source of unwanted side-effects, which is why both DC and CC were used in conjunction.

The models of the MER and the DBS system contain several assumptions and simplifications and the results have to be considered as approximations to reality. The MER stimulation contact for instance is assumed to inject current through the entire surface which might not be totally accurate due to the space occupied by the retracted recording microelectrode at the bottom. The influence is assumed to be irrelevant, but it has not been evaluated yet. Another assumption is that there is no electrode-tissue interface for the MER stimulation contact as the PES for the DBS model. This interface is disregarded due to the small dimensions of the MER system and to the short period of time taken to perform the intraoperative stimulation tests.

Furthermore, Figure 4 shows how each system responds differently to conductivity changes in the surrounding media. For the homogeneous electrical conductivity of 0.123 S/m, both systems reached nearly the same EF extension despite differences in the operating mode and contact dimensions. This specific result responds to the conductivity value used and to the inclusion of a PES with a lower conductivity in the DBS model [19]. On the other hand, when using a lower conductivity in the surrounding medium as for the patient-specific models (~0.079 S/m), the EF volume obtained with the MER lead was considerably larger, e.g., from 23.9 mm^3^ to 49.5 mm^3^ for 1 mA (Figure 3 compared to Figure 5). The magnitude of the EF, dependent on the current density (Equation (1)), increases as the contact surface area decreases [25], and thus the smaller contact of the MER system is capable of generating a similar EF to the larger DBS contacts. For a lower conductivity of the surrounding medium, the system operated under current control has to increase the voltage in order to maintain a constant current through the contact.

The fast technical development and the introduction of new supportive systems for DBS surgery along the improved imaging facilities are making direct targeting more common [29]; however, the selection of the stimulation parameters is still driven by the clinical outcome. In this regard, computer models represent a valuable aid for the clinical staff due to the possibility to visualize how the stimulation field is influenced by changes in the stimulation parameters and electrical setup.

## 5. Conclusions

Simulations showed that the EF generated by MER stimulation can be partially reproduced with the DBS electrodes. Nevertheless, the present study demonstrated that differences exist due to different electrical settings, operating modes, and electrode designs, resulting in EF deformations and variations in extension and volume. Those results might explain the differences between the intraoperatively identified optimal position and the one induced during chronic DBS. Clinicians should be aware of this and take these influences and differences into account during MER stimulation.

## Figures and Tables

**Table 1:** Summary of the settings to investigate the EF due to differences between the MER stimulation and the DBS contacts.

Investigation	Model	Active Contacts and Stimulation Amplitude	Ground
Grounding the guide tube	2D Axisymmetric	MER stim. contact: 1–4 (mA)	Guide tube or Outer boundaries
Parallel MER leads	3D Homogeneous	MER stim. contact: 1, 3 (mA)	Guide tube
Non-active contacts for DBS	2D Axisymmetric	MER stim. contact: 1, 3 (mA)/DBS C0, C1, C2 or C3: 1, 3 (V)	Guide tube/Outer boundaries
Position of the DBS active contact	3D Patient-specific	MER stim. contact: 1, 3 (mA)/DBS C0 or C1: 1, 3 (V)	Guide tube/Outer boundaries
Clinical case	3D Patient-specific	MER stim. contact: 0.4 mA/DBS C0, C1 or C2: 2, 3 (V)	Guide tube/Outer boundaries
